# Illumination of understudied ciliary kinases

**DOI:** 10.3389/fmolb.2024.1352781

**Published:** 2024-03-08

**Authors:** Raymond G. Flax, Peter Rosston, Cecilia Rocha, Brian Anderson, Jacob L. Capener, Thomas M. Durcan, David H. Drewry, Panagiotis Prinos, Alison D. Axtman

**Affiliations:** ^1^ Structural Genomics Consortium, UNC Eshelman School of Pharmacy, University of North Carolina at Chapel Hill, Chapel Hill, NC, United States; ^2^ Department of Chemistry, University of North Carolina at Chapel Hill, Chapel Hill, NC, United States; ^3^ The Neuro’s Early Drug Discovery Unit (EDDU), McGill University, Montreal, QC, Canada; ^4^ UNC Lineberger Comprehensive Cancer Center, School of Medicine, University of North Carolina at Chapel Hill, Chapel Hill, NC, United States; ^5^ Structural Genomics Consortium, University of Toronto, Toronto, ON, Canada

**Keywords:** kinase, cilia, ciliogenesis, ciliopathy, understudied, chemical probe, cancer, neurological disorder

## Abstract

Cilia are cellular signaling hubs. Given that human kinases are central regulators of signaling, it is not surprising that kinases are key players in cilia biology. In fact, many kinases modulate ciliogenesis, which is the generation of cilia, and distinct ciliary pathways. Several of these kinases are understudied with few publications dedicated to the interrogation of their function. Recent efforts to develop chemical probes for members of the cyclin-dependent kinase like (CDKL), never in mitosis gene A (NIMA) related kinase (NEK), and tau tubulin kinase (TTBK) families either have delivered or are working toward delivery of high-quality chemical tools to characterize the roles that specific kinases play in ciliary processes. A better understanding of ciliary kinases may shed light on whether modulation of these targets will slow or halt disease onset or progression. For example, both understudied human kinases and some that are more well-studied play important ciliary roles in neurons and have been implicated in neurodevelopmental, neurodegenerative, and other neurological diseases. Similarly, subsets of human ciliary kinases are associated with cancer and oncological pathways. Finally, a group of genetic disorders characterized by defects in cilia called ciliopathies have associated gene mutations that impact kinase activity and function. This review highlights both progress related to the understanding of ciliary kinases as well as in chemical inhibitor development for a subset of these kinases. We emphasize known roles of ciliary kinases in diseases of the brain and malignancies and focus on a subset of poorly characterized kinases that regulate ciliary biology.

## 1 Introduction

Kinases are a class of proteins that regulate a diversity of pathways via phosphorylation. Mutations, loss, or overexpression of kinases have each been implicated in various pathologies. Their involvement in essential disease-driving processes makes kinases an attractive protein class for pharmacological manipulation in cancer, neurological disorders, viruses, and other diseases. Another very attractive feature of kinases is that they bind small molecules in their ATP-binding site and thus are highly tractable. The more than 80 FDA-approved small molecule drugs (Roskoski, 2023) support their tractability and provide tangible evidence of the significant success that has been realized when targeting these proteins, especially for cancer. Cancer is just one disease area in which kinases play key roles. It has been suggested that vast therapeutic potential can be realized through targeting this protein class for other disorders in need of more effective therapeutic options (Krahn et al., 2020; Axtman, 2021).

Ciliogenesis is a highly regulated process involving the assembly of cilia extruding from the plasma membrane. There has been a growing pool of knowledge which implicates kinases as regulators of ciliogenesis (Goto et al., 2017). Kinases are also responsive to changes in cilia (Christensen et al., 2012). Some kinases are localized to cilia, while others are nuclear or located within different organelles, but still impact ciliogenesis (Wirschell et al., 2011; Christensen et al., 2012; Abraham et al., 2022). The many facets of cilia biology, including their evolution, architecture, composition, generation, localization, regulation, and subtype characterization have been extensively described by others (Hildebrandt et al., 2011; Lee and Gleeson, 2011; Christensen et al., 2012; Christensen et al., 2017; Goto et al., 2017; Reiter and Leroux, 2017; Modarage et al., 2022; Arora et al., 2023) and thus will not be the focus of this review. Dysfunctional ciliogenesis and/or aberration of other ciliary signaling pathways can result in human ciliopathies, an emerging class of more than 30 disparate, single-gene, developmental and degenerative disorders characterized by defects in ciliary structure and function (Hildebrandt et al., 2011; Lee and Gleeson, 2011; Goto et al., 2017). These typically result from absent or disrupted function of primary cilia. Primary cilia play a role in sensory detection rather than motility, represent signaling nodes, exist on the surface of almost every quiescent, differentiated cell in the human body, and are essential for the development and homeostasis of human tissues (Lee and Gleeson, 2011; Christensen et al., 2012; Goto et al., 2017; Reiter and Leroux, 2017; Loukil et al., 2021). Primary cilia coordinate signaling pathways in cell cycle control, differentiation, migration, neurotransmission, and other key cellular processes, making them critical organelles (Christensen et al., 2012).

While ciliopathies are rare disorders, they often present with shared clinical features. These include cystic kidneys, situs inversus, retinal issues, brain malformation and/or intellectual disability, heterotaxy, hydrocephaly, craniofacial and skeletal abnormalities, liver disease, anosmia, congenital heart diseases and cardiac fibrosis, infertility, improper circulation of cerebral spinal fluid, hypoplasia, obesity, retinal degeneration, blindness, diabetes, tumorigenesis, and polydactyly (Lee and Gleeson, 2011; Wirschell et al., 2011; Christensen et al., 2012; Goto et al., 2017; Villalobos et al., 2019; Arora et al., 2023; Benmerah et al., 2023). From this list it is clear that ciliopathies impact several vital organs including the brain, heart, kidneys, liver, eyes, respiratory tract, and reproductive system as well as digits. Ciliopathies can be quite organ-specific with examples such as polycystic kidney disease, retinitis pigmentosa, and nephronophthisis. They can also be pleiotropic disorders, like cerebello-oculo-renal syndrome, Primary Ciliary Dyskinesia (PCD), Bardet-Biedl syndrome, Meckel-Gruber syndrome, orofaciodigital syndrome 1, Joubert syndrome, STAR syndrome, and Jeune asphyxiating thoracic dystrophy (Zaghloul and Katsanis, 2009; Hildebrandt et al., 2011; Lee and Gleeson, 2011; Wirschell et al., 2011; Guen et al., 2016; Goto et al., 2017; Smith et al., 2022; Benmerah et al., 2023). More comprehensive reviews of ciliopathies provide additional details on their inheritance, genetics, clinical symptoms, and other features (Hildebrandt et al., 2011; Lee and Gleeson, 2011; Christensen et al., 2012; Abraham et al., 2022; Modarage et al., 2022). Advances in genomics, proteomics, next-generation sequencing, and transcriptomics has led to the identification of disease-causative ciliary gene mutations and allowed for a better understanding of the pathogenesis of these disorders (Lee and Gleeson, 2011; Wirschell et al., 2011; Modarage et al., 2022). Despite these advances, difficulties persist in diagnosing ciliopathies that present with similar phenotypes (Modarage et al., 2022).

Primary cilia also play important roles in brain development and are involved in patterning, maintenance, and proliferation of the progenitor pool (Youn and Han, 2018; Rocha and Prinos, 2022). These organelles influence neuronal differentiation, connectivity, and activity (Lepanto et al., 2016; Bowie and Goetz, 2020; Rocha and Prinos, 2022). Mutations in some ciliary genes are associated with brain abnormalities and may result in neurological manifestations. Examples of neuronal developmental disorders that result, in part, from ciliary dysfunction include autisms and Joubert syndrome, while Parkinson’s disease (PD) and amyotrophic lateral sclerosis (ALS) are examples of neurodegenerative diseases in which aberrant cilia are involved (Doherty, 2009; Trulioff et al., 2017; Youn and Han, 2018; Ma et al., 2022; Rocha and Prinos, 2022).

Various cancer cells have been shown to lack cilia expression (Menzl et al., 2014; Cao and Zhong, 2016; Higgins et al., 2019). Changes in primary cilia have been noted in renal, prostate, cholangiocarcinoma, pancreatic, skin, brain and breast cancers (Seeley et al., 2009; Basten et al., 2013; Basten and Giles, 2013; Hassounah et al., 2013; Seeger-Nukpezah et al., 2013; Menzl et al., 2014; Snedecor et al., 2015; Higgins et al., 2019). Renal epithelial and human primary melanoma cells demonstrate significant losses of cilia in response to carcinogens (Basten et al., 2013; Snedecor et al., 2015; Higgins et al., 2019). Pancreatic cancer cells and intraepithelial neoplasia lesions from human pancreatic ductal adenocarcinoma demonstrate significantly suppressed ciliogenesis (Seeley et al., 2009; Emoto et al., 2014; Higgins et al., 2019). Many of the examples above link loss of cilia to cancer, but this is not always the case. There is evidence suggesting the existence of primary cilia could be a hallmark of aggressive pancreatic ductal adenocarcinoma (Emoto et al., 2014). Moreover, medulloblastomas with activation in Hedgehog (Shh) or Wnt signaling were found to have primary cilia, but primary cilia were not found in medulloblastomas in other molecular subgroups (Han et al., 2009).

Changes in cilia, while not fully understood, have long been associated with cancer development and progression. Using agents that inhibit or promote ciliogenesis has been considered as a therapeutic approach for different cancers (Liu et al., 2018; Lee et al., 2021). An established interaction between cilia and autophagy, a cellular clearance mechanism, has provided a hypothesis for at least one essential process that is disrupted when cilia are lost in cancer (Cao and Zhong, 2016). The role of cilia in suppressing abnormal cell proliferation through regulating cell cycle entry/exit is another key pathway disrupted when cilia are absent (Cao and Zhong, 2016). Links between primary cilia and important signaling pathways with cancer implications, such as Hedgehog, Wnt, and Notch, have been extensively reviewed (Goetz et al., 2009; Egeberg et al., 2012; Seeger-Nukpezah et al., 2013; Liu et al., 2018; Higgins et al., 2019; Mill et al., 2023) and will not be the focus herein.

## 2 Ciliary kinases

Embedded in the disease-causative ciliary networks are many regulatory kinases that represent druggable nodes. While some of these kinases have been the focus of drug discovery campaigns, others have not been investigated. These “understudied” kinases have had significantly less effort devoted to their study and thus their functions remain poorly annotated in the literature. Still, there are described links between understudied kinases and ciliary function. We provide herein a discussion of three subfamilies of understudied kinases that play a role in ciliary function and/or in pathways that result in ciliopathies. Furthermore, we provide a summary of tool molecules that can be used in efforts to study these ciliary kinases.

**FIGURE 1:**
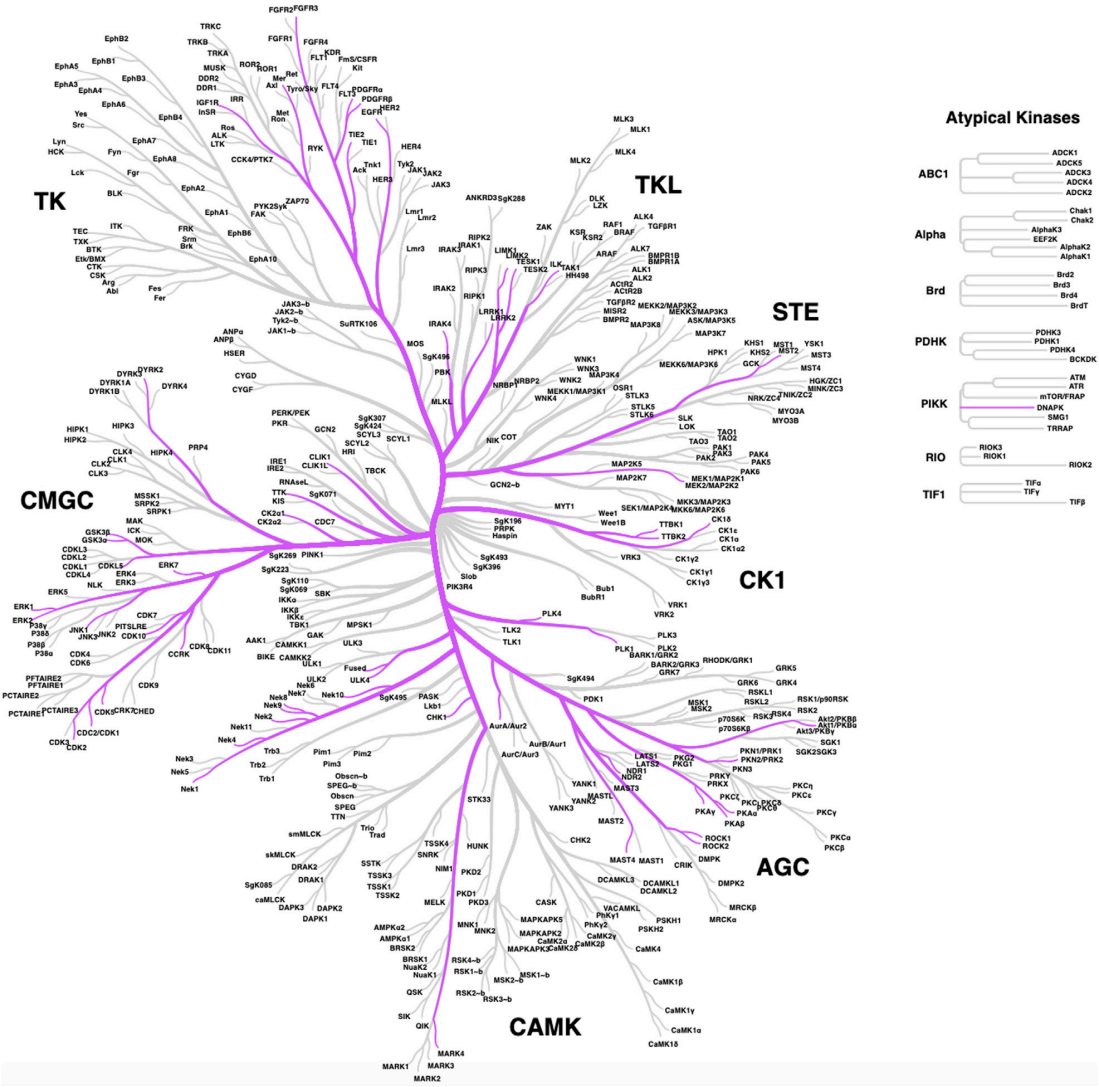
Distribution of ciliary kinases on the phylogenic tree of the human kinome. The same kinases included in Table 1 are shown except for lipid kinases PIPK1γ and PIK3CB, which are not pictured here.

Since many human kinases, both well-studied and understudied, have described roles in ciliary pathways, a subset has been explored as potential targets for pharmacological intervention in the pursuit of therapies for patients that suffer from ciliopathies or other conditions, such as cancer or neurodegeneration. We provide a list of human kinases with reported links to ciliary pathways and dysfunction in Table 1. This table specifies the described roles of each kinase in cilia, which includes the following categories: ciliary signaling, ciliary resorption, ciliary function, ciliary motility, ciliogenesis, cilia length, ciliary trafficking, ciliary stability, ciliary structure, ciliary localization, and ciliary dynamics. These categories better define the ciliary function(s) of each of the kinases in Table 1. While our list is extensive, it is not a compendium of all human kinases that regulate cilia. The distribution of these kinases around the human kinome tree (Figure 1) highlights the involvement of disparate kinases in ciliary function(s) and demonstrates that these kinases are not clustered in a single family. Both Table 1; Figure 1 reinforce the idea that kinases are ubiquitous in ciliary pathways and play an indispensable role in their generation, structure, stability, resorption, and function.

**TABLE 1:** Selected kinases and their reported links to cilia formation and dysfunction. References are provided for further reading on these kinases. Additional details are provided in the following sections for CDKL, NEK, and TTBK family members as well as LRRK2, PLK1, CDK20/CCRK, CILK1/ICK, CDK10, PKN2, MAPK15, STK38L, STK36, and ULK4.

Kinase	Family	Ciliary link	PMID(s)
AKT1	AGC	Ciliary signaling	27638178
MAST4	AGC	Ciliary resorption	37726137
PKA	AGC	Ciliary function and motility	22007132; 21513695; 34582081
PKN2	AGC	Ciliogenesis	34582081; 27104747
ROCK1	AGC	Ciliogenesis	33392209; 32663194
ROCK2	AGC	Ciliogenesis	33392209; 32663194
STK38L/NDR2	AGC	Ciliogenesis and cilia length	34485842; 23435566; 20887780; 30135513; 30714141; 30108113; 29108249
DNAPK/PRKDC	Atypical	Ciliogenesis	30867219; 33462409
CHK1	CAMK	Ciliogenesis	30867219
MARK4	CAMK	Ciliogenesis	23400999
CK1D	CK1	Ciliogenesis	24648492
TTBK1	CK1	Ciliogenesis	37558899; 37059819
TTBK2	CK1	Ciliogenesis	31934864; 37059819; 24982133; 30532139
CDK1	CMGC	Ciliogenesis and ciliary resorption	23345402
CDK10	CMGC	Ciliogenesis and cilia length	27104747; 29130579; 34582081; 24218572; 28886341; 32127582; 28178678
CDK2	CMGC	Motile ciliogenesis	30152757
CDK20/CCRK	CMGC	Ciliogenesis, ciliary signaling and cilia length	34624068; 35609210; 31506943; 32317081; 34582081; 37469151; 19672860; 25500144; 28817564; 17565152; 23743448
CDK5	CMGC	Cilia length	27053712
CDKL5	CMGC	Ciliogenesis and cilia length	29420175; 34624412; 37490324; 37084253
CILK1/ICK	CMGC	Ciliogenesis, ciliary function and cilia length	31506943; 32732286; 34582081; 24853502; 19185282; 27466187; 24797473
CK2A1	CMGC	Ciliary trafficking and stability	33846249
DYRK2	CMGC	Ciliogenesis	34582081; 32758357
ERK1/MAPK3	CMGC	Ciliogenesis and cilia length	36914265
ERK2/MAPK1	CMGC	Ciliogenesis and cilia length	36914265
GSK3⍺	CMGC	Ciliogenesis	30867219
GSK3β	CMGC	Ciliogenesis	30867219; 17450132
JNK1	CMGC	Ciliogenesis and ciliary function	37851005
PIK3CB	Lipid	Ciliogenesis	30867219
PIPK1γ	Lipid	Ciliogenesis and ciliary signaling	34162535
MAPK15/ERK7	MAPK	Ciliogenesis, ciliary function and trafficking	29021280; 28745435; 25823377; 34638386; 36266944
AURKA	Other	Ciliogenesis and ciliary resorption	34582081; 27669693; 18381407; 36924208; 29141582; 17604723; 34944109; 17604723
NEK1	Other	Ciliogenesis and ciliary structure	34582081; 16280549; 18387364; 16267153; 10618398; 18533026
NEK10	Other	Ciliogenesis, ciliary signaling and cilia length	29581457; 32414360; 31959991
NEK2	Other	Ciliogenesis and ciliary resorption	34582081; 26493400; 26290419; 16203858; 9647649; 29141582
NEK4	Other	Ciliary stability	21685204; 25798074; 26124960
NEK8	Other	Ciliary localization	21506742; 37598857; 16280549; 16267153; 23973373; 26967905; 18199800; 15872312; 22106379; 34078910
NEK9	Other	Ciliary function and resorption	34582081; 22818914; 12101123; 16079175; 24921005; 21642957; 36712877; 30594554; 27153399; 20562859; 26908619
PLK1	Other	Ciliary resorption and dynamics	34582081; 22701722; 27669693; 23345402
PLK4	Other	Ciliogenesis	26701933; 36924208
STK36/Fused	Other	Ciliogenesis, ciliary structure and localization	36989043; 23907739; 19305393; 16055717; 24284070; 27300315; 21746835; 27445138; 37584603; 34463328
TTK/MPS1	Other	Ciliogenesis and ciliary resorption	27669693
ULK4	Other	Ciliogenesis	27445138; 36989043
MEK2/MAP2K2	STE	Ciliogenesis	30867219
STK3/MST2	STE	Ciliogenesis	25367221
EGFR	TK	Ciliary function	27638178; 30867219
FGFR3	TK	Cilia length	34582081; 27638178
IGF1R	TK	Ciliogenesis and ciliary signaling	27638178
MERTK	TK	Ciliogenesis	30867219
PDGFR⍺	TK	Ciliogenesis and ciliary signaling	27638178
PDGFRβ	TK	Ciliogenesis	30867219
TIE2	TK	Ciliary signaling	27638178
IRAK4	TKL	Ciliogenesis	30867219
LIMK2	TKL	Ciliogenesis	25849865
LRRK2	TKL	Ciliogenesis	36924208; 30867219
TAK1	TKL	Ciliary dynamics	27638178; 30867219
TESK1	TKL	Ciliogenesis	25849865

Kinase activity is essential for ciliary activity and, thus, kinase dysfunctions are responsible for a plethora of pathological conditions, including neurological disorders and cancer. In the next subsection examples of important ciliary kinases associated with neuronal tissue in health and disease will first be reviewed. We then provide a summary of how ciliary kinases are hijacked by cancers to drive oncogenic processes.

### 2.1 Ciliary kinases with roles in neurodevelopment and neurological diseases

Several more studied and a few lesser studied kinases have connections to neuronal development and disorders. While we will introduce the roles of members of the CDKL, NEK, and TTBK families in these processes in the next section, examples of other kinases from Table 1 with to that modulate ciliary function in the brain are included below. Table 2 serves as a quick summary of the ciliary kinases from Table 1 that have been linked to neurological issues and which are discussed in some detail herein.

**TABLE 2:** Subset of ciliary kinases from Table 1 with links to neurodevelopment, neurological disorders, or cancer. Further reading on each of these kinases is provided in various subsections.

Kinases with role(s) in neurodevelopment and/or neurological disease	Kinase with role(s) in cancer
LRRK2	CDK20/CCRK
PLK1	CILK1/ICK
CDKL5	CDK10
NEK1	PKN2
TTBK1	MAPK15
TTBK2	STK38L
	STK36/Fused
	ULK4
	NEK1
	NEK2
	NEK4
	NEK8
	NEK9
	NEK10

#### 2.1.1 LRRK2

Cilia alterations are implicated in neurodegenerative disorders such as Parkinson’s disease (PD). Leucine-rich repeat kinase 2 (*LRRK2*) encodes a kinase that is involved in vesicular membrane trafficking and is one the genes mutated in familial cases of Parkinson’s disease (Alessi and Sammler, 2018). Primary cilia status has been assessed in mouse and human studies of PD. Cilia loss was detected in neurons and astrocytes from mice carrying the G2019S familial *LRRK2* mutation and was associated with dysregulation of Hedgehog signaling (Khan et al., 2021). The G2019S *LRRK2* mutation was also found to result in primary cilia loss in iPSC (induced pluripotent stem cell) derived neurons from PD patients (Dhekne et al., 2018). Many small molecule inhibitors have been developed for LRRK2 (Azeggagh and Berwick, 2022), several of which have advanced into clinical trials for PD (Kingwell, 2023).

#### 2.1.2 PLK1

Polo-like kinase (PLK1) localizes to the primary cilium transitional zone and is activated during cilia disassembly (Seeger-Nukpezah et al., 2012). In NIH3T3 cells, PLK1 is recruited to the pericentriolar matrix by PCM1 (pericentriolar material 1), leading to cilia disassembly by activating HDAC6 prior to mitosis entry (Wang et al., 2013). The interaction between PCM1 and PLK1 is dependent on the phosphorylation of PCM1 by CDK1 (cyclin-dependent kinase 1) (Wang et al., 2013). PLK1 interacts with Treacle (encoded by *TCOF1* gene), which is a centrosome and kinetochore associated-protein important for mitotic progression and proper neurogenesis (Sakai et al., 2012). Treacle interacts with Plk1 and promotes proliferation in the developing cortex of mice, demonstrating that Plk1 is necessary for neural progenitor mitotic progression (Sakai et al., 2012).

### 2.2 Ciliary kinases hijacked to propagate cancer

Ciliary kinases regulate signaling pathways such as Hedgehog, Wnt, Hippo, and other pathways that are functionally linked to ciliogenesis and cancer. Many of the ciliary kinases discussed below are deregulated in tumors. Herein we will focus on a subset of ciliary kinases from Table 1 that are linked to cancer with the aim to promote efforts to understand their function. Table 2 provides a quick reference of the ciliary kinases from Table 1 with identified roles in cancer propagation and/or progression, all of which are highlighted in more detail in the following sections.

#### 2.2.1 CDK20/CCRK

Cyclin-dependent kinase 20 (CDK20), or more commonly referred to as cell cycle-related kinase (CCRK), is a member of the CDK family. CDK20/CCRK has been functionally linked to cell cycle checkpoint control, is essential for cell proliferation, and is centrally involved in the development of many malignancies (Chivukula and Malkhed, 2023). Several studies have reported the overexpression of CDK20 in cancers from the brain, colon, liver, lung, and ovary (Chivukula and Malkhed, 2023). CDK20 upregulation in some of these cancers is clinically significant as it correlates with tumor staging, shorter patient survival, and poor prognosis (Wu et al., 2009; Feng et al., 2015). Downstream of Smo and upstream of Gli, CDK20 regulates ciliogenesis and Hedgehog signaling across organisms from *Chlamydomonas* to *C. elegans* to humans (Snouffer et al., 2017). CDK20 has been functionally linked to glioblastoma where it behaves as an oncogene and contributes to increased proliferation (Ng et al., 2007). Glioblastoma cells display deregulated, high levels of CDK20, and its depletion inhibits glioblastoma cell proliferation in a cilium-dependent manner (Yang et al., 2013). In this context, the effects of CDK20 on ciliogenesis were found to be mediated by its substrate, intestinal cell kinase (ICK) (Yang et al., 2013). Depletion of CDK20 leads to accumulation of ICK at ciliary tips, altered ciliary transport, and inhibition of cell cycle re-entry in NIH3T3 fibroblasts (Yang et al., 2013). All of this evidence makes CDK20 a promising drug target. The CDK20 protein structure was recently predicted using AlphaFold. This structure was used in AI-accelerated hit discovery for CDK20 to produce a novel small molecule inhibitor of CDK20 (Ren et al., 2023).

#### 2.2.2 CILK1/ICK

Ciliogenesis associated kinase 1 (CILK1), previously known as intestinal cell kinase (ICK), is now recognized as a ubiquitously expressed member of the RCK family of serine/threonine kinases (Fu et al., 2019). Inactivating loss-of-function mutations in the human *CILK1* gene produce lethal developmental ciliopathies, namely, the endocrine-cerebro-osteodysplasia (ECO) syndrome (MIM 612651) (Lahiry et al., 2009) and short-rib polydactyly syndrome (SRPS) type II (MIM 263520) (Paige Taylor et al., 2016). In mice, both Cilk1 knock-out and Cilk1 knock-in mutations have recapitulated human ciliopathies. CILK1 has a fundamental role in the function of cilia, and it is required for ciliogenesis (Chaya et al., 2014) by controlling ciliary length (Moon et al., 2014). CILK1 regulates the ciliary localization of Shh pathway components and the localization of intraflagellar transport (IFT) components at ciliary tips (Chaya et al., 2014). CILK1 is activated by phosphorylation at Thr157 by its upstream kinase CDK20, which triggers its autophosphorylation at Tyr159. CILK1 phosphorylates Raptor, Scythe, GSK3β, and KIF3A, linking CILK1 function to cellular metabolism, ciliogenesis, and Hedgehog signaling (Fu et al., 2019). KIF3A, a kinesin motor protein, controls IFT anterograde transport thus raising the possibility that effects of CILK1 on ciliogenesis are mediated through its phosphorylation of KIF3A. Through phosphorylation of these substrates, CILK1 is involved in the regulation of mTOR, Wnt, Hedgehog, and FGFR signaling pathways. Interestingly, CILK1/ICK was found to mediate the effect of CDK20 on ciliogenesis in glioblastoma cells (Yang et al., 2013) and it also regulates the inhibitory effect of fibroblast growth factor on cilia by interacting with FGFR3 (Kunova Bosakova et al., 2019). Thus, ICK seems to be a signaling hub that integrates multiple signaling pathways in ciliary control (Fu et al., 2019).

#### 2.2.3 CDK10 and PKN2

CDK10 is a kinase of the CDK family that forms a heterodimer with Cyclin M (Guen et al., 2013; Guen et al., 2017). The heterodimer promotes cell proliferation via phosphorylating the oncogene ETS2 and is an important regulator of triple negative breast cancer response to endocrine therapy (Iorns et al., 2008). CDK10/cyclin M regulates ciliogenesis through phosphorylation of PKN2 as well as regulating RhoA and the actin cytoskeleton. Mutations in CDK10/Cyclin M cause STAR syndrome, a developmental disorder affecting the skeleton and limbs (Guen et al., 2013). Homozygous mutations in CDK10 cause Al Kaissi syndrome, a neurodevelopmental disorder displaying growth retardation and spine and craniofacial malformations (Windpassinger et al., 2017). Cells from Cdk10/Cyclin M deficient mice or STAR mutants were reported to present with elongated cilia (Guen et al., 2016; Windpassinger et al., 2017). CDK10 phosphorylates PKN2, a kinase that is also critical for ciliogenesis. This suggests that the effects of CDK10 on cilia are mediated, in part, by PKN2 (Guen et al., 2016). PKN2 also regulates RhoA cytoskeleton stress fiber dynamics and cell migration (Guen et al., 2016). Because PKN2 has been linked to several types of cancer including breast, colorectal, renal, head and neck and prostate cancers, it is considered an emerging target (Patel et al., 2020). Dihydropyrrolopyridinone-based PKN2 chemical tools that could enable studies around this kinase were reported in the last few years (Scott et al., 2020; Scott et al., 2022). More recently, computational docking approaches identified promising chemical leads for PKN2 (Al-Sha’er et al., 2023). Although there are not yet specific CDK10 inhibitors, recent efforts toward identifying small molecules targeting CDK10 are underway (Robert et al., 2020).

#### 2.2.4 MAPK15 (aka ERK7/ERK8)

MAPK15, originally known as ERK7/ERK8, is an atypical member of the MAPK kinase family. It is an understudied MAP kinase as its functions have only recently started to be elucidated (Deniz et al., 2023). MAPK15 is functionally implicated in a variety of cellular activities such as cell proliferation, apoptosis, autophagy, and maintenance of genomic integrity (Deniz et al., 2023). This kinase also plays an evolutionarily conserved, essential role in ciliogenesis (Kazatskaya et al., 2017). Knockdown of MAPK15 diminishes the number and the length of cilia in *X. laevis*, *C. elegans*, and human neurons (Miyatake et al., 2015). In addition, MAPK15 regulates the localization of ciliary proteins involved in cilium structure, transport, and signaling and regulates apical body migration by phosphorylating CAPZIP (Miyatake et al., 2015; Kazatskaya et al., 2017). MAPK15 has been functionally linked to the Shh subgroup of medulloblastoma where it regulates Hedgehog signalling and tumorigenesis in a cilia-dependent fashion (Pietrobono et al., 2021).

#### 2.2.5 STK38L

STK38L, also known as NDR2 kinase, is a member of the nuclear Dbf2-related (NDR) serine/threonine kinase family. The NDR protein kinases play crucial roles in the control of cell proliferation, apoptosis, and morphogenesis (Santos et al., 2023). These kinases are also important regulators of the Hippo signaling pathway through phosphorylating YAP/TAZ (Hergovich, 2016). STK38L/NDR2 is involved in primary cilium formation (Chiba et al., 2013) and is mutated in a naturally occurring canine ciliopathy, termed early retinal degeneration (Goldstein et al., 2010). Stk38l deletion in mice caused decreased proliferation of retinal amacrine cells by increasing the expression of neuronal stress genes while decreasing the expression of synaptic genes (Léger et al., 2018). STK38L is crucial for ciliogenesis via phosphorylating Rabin8 and impairing pre-ciliary membrane biogenesis at the pericentrosome (Chiba et al., 2013). Genetic variants in STK38L have been associated with increased glioma risk (Chen et al., 2019) and coding mutations were reported in microsatellite-unstable colorectal cancer (Kondelin et al., 2018). STK38L was reported to be overexpressed in KRAS-dependent pancreatic cancer cell lines where it was found to be essential for cell proliferation (Grant et al., 2017).

#### 2.2.6 STK36/Fused

STK36/Fused is a member of the serine/threonine protein kinase (STK) family. This kinase is similar to a *Drosophila* protein, Fused, that plays a key role in the Hedgehog signaling pathway by regulating the activity of Gli transcription factors by promoting their nuclear localization and opposing the effect of Suppressor of Fused (SUFU) (Murone et al., 2000). STK36 is required for postnatal development through regulating the homeostasis of cerebrospinal fluid (CSF) and ciliary function. STK36 was shown to be essential for the construction of the central pair apparatus of motile cilia (Wilson et al., 2009). Mutation of STK36 leads to primary ciliary dyskinesia with a central pair defect (Edelbusch et al., 2017). Knockout of the homologous mouse gene leads to severe growth retardation and congenital hydrocephalus due to a functional defect in motile cilia (Merchant et al., 2005). The STK36 effects on ciliogenesis and CSF flow are functionally linked to its binding to ULK4, which is another kinase indispensable for motile ciliogenesis (Zhang et al., 2023).

#### 2.2.7 ULK4

Unc51-like kinase 4 (ULK4) belongs to the Unc-51-like serine/threonine kinase (STK) family and encodes a pseudokinase with unclear function (Luo et al., 2022). It is a paralogue of STK36, which is also involved in ciliogenesis. ULK4 has known roles in the remodeling of cytoskeletal components, and it regulates neurite branching and elongation as well as neuron cell motility (Lang et al., 2014). Accumulating evidence indicates that ULK4 participates in corticogenesis, cilia maintenance, myelination, and white matter integrity (Lang et al., 2014). Ulk4 deletion in mice causes decreased intermediate neural progenitors and increased apoptosis, thus disrupting normal cortical development (Liu et al., 2016a). Likewise, Ulk4 null knockout mice present disturbed motile cilia development and disorganized ciliary beating, which impairs CSF flow eventually leading to congenital hydrocephalus (Vogel et al., 2012; Liu et al., 2016b). These phenotypes are identical to STK36 hypomorphic mutants, supporting that ULK4 and STK36 interact as part of a complex (Zhang et al., 2023). The ULK4 protein contains a pseudokinase domain at the N-terminus and is predicted to be catalytically inactive. Its pseudokinase domain interacts with STK36, indicating that ULK4 can directly regulate active kinases despite being catalytically inactive itself (Zeqiraj and van Aalten, 2010). The structure of ULK4 has been resolved, enabling virtual and experimental screens that have identified promising chemical scaffolds for efforts to design specific ULK4 inhibitors (Khamrui et al., 2020). Functional genomic analysis identified the master transcription factor Foxj1 and Foxj1 pathway in cilia as well as an array of other ciliogenesis factors are specifically regulated by ULK4 (Liu et al., 2016a). Furthermore, ULK4 was recently reported to be a component of primary cilia in the neuroepithelium where it acted as a positive regulator of Shh signaling (Mecklenburg et al., 2021). Altogether, these studies demonstrate that ULK4 plays a vital role in ciliogenesis and that deficiency of ULK4 causes hydrocephalus and other ciliopathy-related phenotypes prevalent in neurodevelopmental and neuropsychiatric disorders (Luo et al., 2022).

## 3 Understudied ciliary kinase families

**FIGURE 2:**
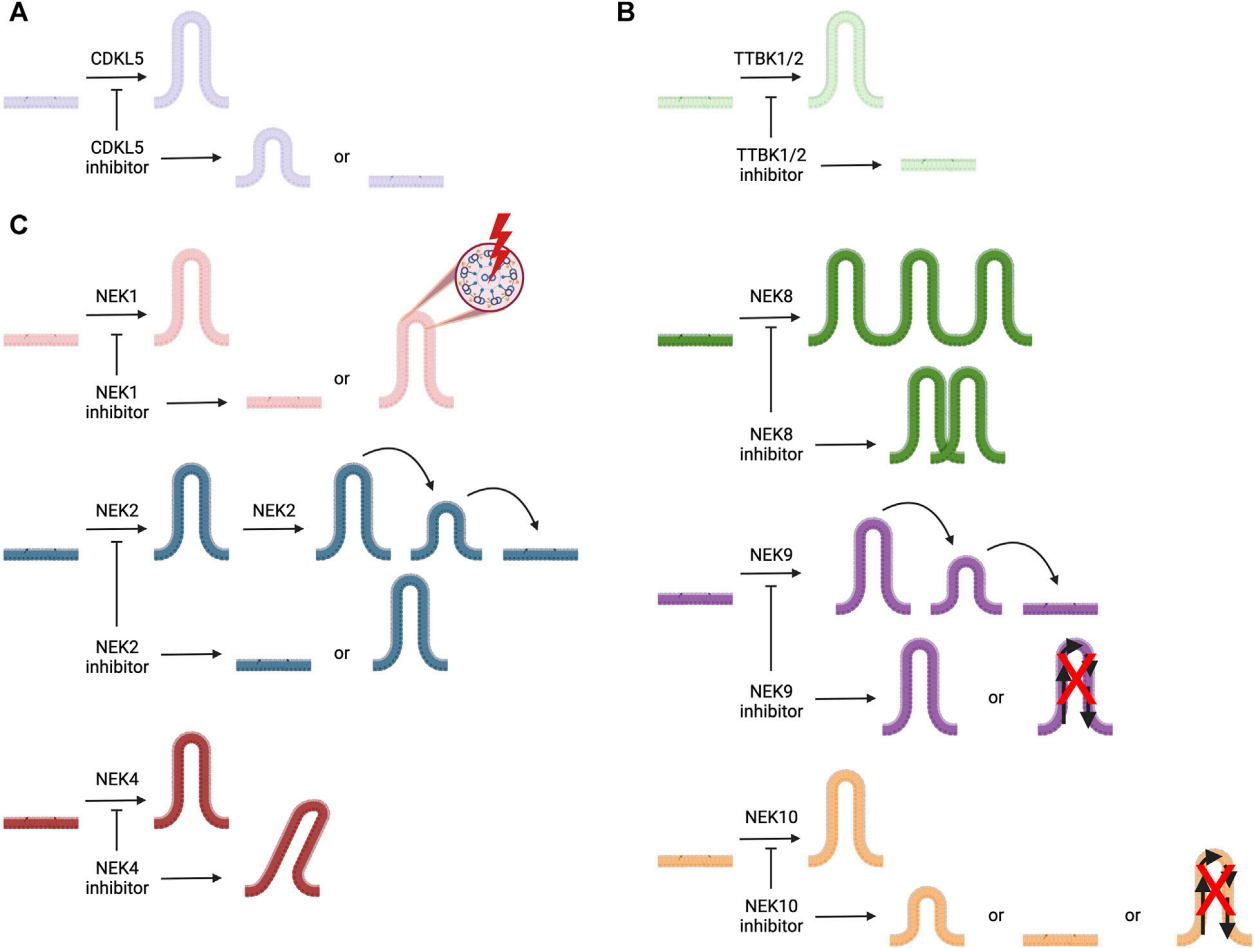
Illustration of cilia, represented in different colors for each kinase, the roles of ciliary kinases CDKL5, NEK1, NEK2, NEK4, NEK8, NEK9, NEK10, TTBK1, and TTBK2, and how inhibition of these kinases is expected to impact ciliary morphology and/or function(s). **(A)** CDKL5 is involved in ciliogenesis and regulating cilia length. **(B)** TTBK1 and TTBK2 both play a role in ciliogenesis. **(C)** NEK1 regulates ciliogenesis and ciliary structure; NEK2 plays a role in ciliogenesis and ciliary resorption; NEK4 helps to stabilize cilia; NEK8 is involved with ciliary localization; NEK9 regulates ciliary function and its resorption; and NEK10 modulates ciliogenesis, ciliary signaling, and cilia length.

The primary literature provides insights into the roles of the kinases in Table 1, including those related to cilia and in the pathology of ciliopathies as well as other diseases. In general, fewer papers have been dedicated to certain families of kinases. In the sections that follow, we provide an in-depth discussion of the roles of understudied members of the cyclin-dependent kinase like (CDKL), never in mitosis gene A (NIMA) related kinase (NEK), and tau tubulin kinase (TTBK) families as they relate to cilia. To spur research on the ciliary pathway functions and regulation by these understudied kinases, we provide more details about what has been published that connects kinases within these families to cilia. Furthermore, we provide structures of and associated references for the best available chemical probes or high-quality inhibitors of these understudied ciliary kinases that can be used in follow-up studies. Figure 2 illustrates the ciliary pathways driven by members of these understudied kinase families and highlights the expected consequences of kinase inhibition on cilia.

### 3.1 CDKL family

CDKL kinases are a family of five relatively underexplored serine/threonine human kinases: CDKL1, CDKL2, CDKL3, CDKL4, and CDKL5. This family has the highest sequence similarity to cyclin-dependent kinases (CDKs) (Canning et al., 2018; Ong et al., 2023). CDKLs contain a cyclin binding domain, although no cyclin-dependent functions have been ascribed. While little is known about CDKL1–4 regarding their function and role(s) in human biology, CDKL5 has been identified as a regulator of ciliogenesis and cilia length (Canning et al., 2018). Structural characterization of all CDKLs, except for CDKL4, has confirmed them to contain a conserved N-terminal kinase domain with variable C-termini (Canning et al., 2018). The C-termini of CDKL2 and CDKL3 were found to have an atypical αJ helix necessary for their catalytic functions, which is absent in CDKL1 and CDKL5 (Canning et al., 2018). Notably, for CDKL5, the C-terminus is involved in trafficking CDKL5 to its subcellular compartments during various developmental stages (Rusconi et al., 2008; Canning et al., 2018; Ong et al., 2023).

CDKL5, also known as serine/threonine kinase 9 (STK9), is the only member of the CDKL family with strong connections to ciliogenesis in humans. CDKL5 is ubiquitously expressed in human tissues, but is observed at higher levels in the hippocampus, cerebellar, striatum, and cortex regions of the CNS in humans and mice, consistent with its roles in dendritic spine growth, brain development, and excitatory synapse composition (Rusconi et al., 2008; Fagerberg et al., 2014; Canning et al., 2018; Ong et al., 2023). A study using RNAi targeting CDKL5, which caused reduced neurite growth and dendritic arborization in rat cortical neurons, confirmed that this kinase regulates neuronal morphogenesis (Chen et al., 2010).

CDKL5 localizes to the basal body of primary cilia, and when aberrantly overexpressed, can lead to jeopardized ciliogenesis (Canning et al., 2018). Hippocampal neurons from Cdkl5 deficient mice presented elongated primary cilia, however no changes in the levels of Wnt and Shh proteins were visualized by Western blot (Di Nardo et al., 2022). Missense mutations in the *CDKL5* gene leads to a syndrome known as CDKL5-deficiency disorder (CDD). CDD is characterized by severe encephalopathy, noncanonical developmental physiology, and intellectual disability with intractable epilepsy onset from early age, reflective of a significant impact on brain function (Olson et al., 2019; Di Nardo et al., 2022; Castano et al., 2023; Ong et al., 2023).

**FIGURE 3:**
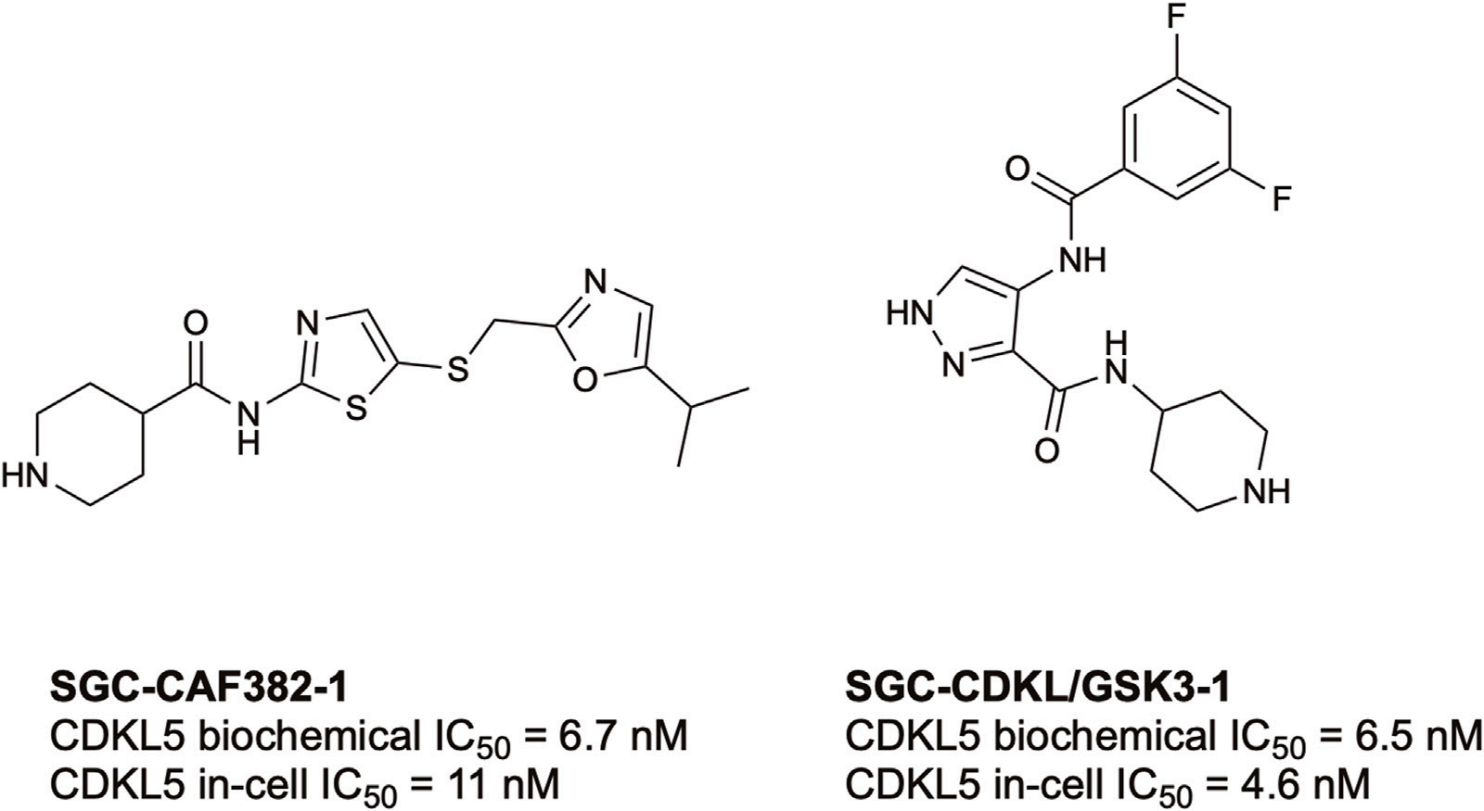
Structures and available potency data for the most advanced CDKL5 chemical probes, SGC-CAF382-1 and SGC-CDKL5/GSK3-1.

To date, there have only been two high-quality chemical probes published by members of the Structural Genomics Consortium (SGC) for CDKL5 (Figure 3). SGC-CAF382-1 (B1 in the original publication) demonstrates a cell-free CDKL5 enzymatic IC_50_ = 6.7 nM and engages with CDKL5 in cells with an IC_50_ = 11 nM (Castano et al., 2023). SGC-CDKL5/GSK3-1 is a CDKL5 and GSK3 (GSK3⍺ and GSK3β) probe with a cell-free CDKL5 enzymatic IC_50_ = 6.5 nM and a nearly equal in-cell CDKL5 target engagement IC_50_ = 4.6 nM (Ong et al., 2023).

CDKL1, the only other CDKL kinase with connections to ciliogenesis, has only been studied in model systems (zebrafish and *C. elegans*). The human and *C. elegans* CDKL1 proteins share 77% identity, and both have similar phosphorylation regulatory sites: Thr^14^, Tyr^15^, and Thr^161^ in humans and Ser^14^, Tyr^15^, and Thr^159^ in zebrafish (Hsu et al., 2011). CDKL1, however, has been shown to influence cilia length and Hedgehog signaling in zebrafish, a pathway that is also linked to primary cilia (Hsu et al., 2011; Bangs and Anderson, 2017; Park et al., 2021). In lieu of a high-quality chemical tool for CDKL1, genomics, siRNA, and other techniques have been used to begin to uncover the functions of this kinase. In *C. elegans*, CDKL1 localizes to the transition zone at the base of the cilium, in a CEP-290-dependent manner, and regulates the length of the growing cilia by interacting with IFT anterograde kinesin motor proteins involved in axoneme formation of the cilia (Li et al., 2016; Park et al., 2021). Due to the high sequence identity, known roles of orthologs, and strong connection between Hedgehog signaling and primary cilia, it is proposed that CDKL1 might have an underlying role in human ciliogenesis (Bangs and Anderson, 2017).

### 3.2 NEK family

The NIMA-related kinase family comprises 11 relatively understudied kinases (NEK1–NEK11) that play roles in many important biological processes and have been linked to several diseases. Recent reviews discuss their biological and disease relevance and provide information about inhibitors (Fry et al., 2012; Meirelles et al., 2014; Wells et al., 2018; Pavan et al., 2021; Panchal and Evan Prince, 2023b; Nguyen et al., 2023). These and other reviews highlight that mutations in all NEK family members have been identified in different cancers and the functions of each NEK in cancer progression delineated (Moniz et al., 2011; Panchal and Evan Prince, 2023a). NEK1 mutations have also been identified as a genetic cause of amyotrophic lateral sclerosis (Kenna et al., 2016). Members of the NEK family play various roles in ciliogenesis and are implicated in distinct ciliopathies (Table 1). Statistical analysis has linked the evolution of the NEKs and centrioles, both of which are responsible for organization of microtubule development as well as the basal bodies of cilia (Quarmby and Mahjoub, 2005).

NEK1 is localized to the basal body region and centrosomes (Shalom et al., 2008). Nek1 mutant mice exhibited a range of defects including progressing PKD (Upadhya et al., 2000), which has a well-established tie to ciliary dysfunction (White and Quarmby, 2008). NEK1 serves as a coordinator between ciliogenesis and cell cycle progression, a role that is speculated to involve signaling between the nucleus and the primary cilium (White and Quarmby, 2008). Remarkably, both overexpression of NEK1 and the complete removal of NEK1 severely decreased the percentage of cells bearing a primary cilium, suggesting that ciliogenesis depends on tightly regulated NEK1 expression (Shalom et al., 2008). The authors proposed that this observation could be due to either NEK1 blocking the upregulation of ciliary proteins or due to it physically interfering with the formation of complexes at the centrosome (White and Quarmby, 2008). NEK1 has also been shown to bind KIF3A, a kinesin motor protein crucial for ciliogenesis, further underscoring its role in directly impacting IFT (Quarmby and Mahjoub, 2005; White and Quarmby, 2008).

While fewer papers have been written about its role in ciliogenesis, NEK2 has a confirmed function in cilia homeostasis. NEK2 phosphorylates C-Nap1, Cep68, and Rootletin at the beginning of mitosis, allowing for the formation of the mitotic spindle (Fry et al., 1998; Bahe et al., 2005). This kinase is required for centriole splitting and is involved in the HDAC6 pathway, which promotes tubulin deacetylation (Endicott et al., 2015; DeVaul et al., 2017). NEK2 has been specifically shown to phosphorylate KIF24, ensuring that cilia resorption occurs prior to mitosis, and increased levels of NEK2 facilitate cilia depolymerization (Mirvis et al., 2018). Conversely, reduction of NEK2 leads to centriole defects and deficiencies in cilia biogenesis (Endicott et al., 2015).

NEK4 is localized to the base of primary cilium in RPE cells, suggesting that its role may include cilia stabilization (Basei et al., 2015). This kinase interacts with RPGRIP1 and RPGRIP1L, two proteins associated with ciliopathies (Patnaik et al., 2015). In a study from Coene et al., NEK4 knockdown resulted in a decrease in ciliated cells. The authors hypothesized that the interaction between RPGRIP1/RPGRIP1L and NEK4 forms a scaffold for the assembly of cilium-related kinases and substrates (Coene et al., 2011).

NEK8 is involved in the regulation of ciliary physiology and associated human ciliopathies (Choi et al., 2013; Grampa et al., 2016; Claus et al., 2022). In a mouse model of juvenile cystic kidney disease, mice bearing mutations in Nek8 display defects in ciliary localization that were potentially causal in the emergence of nephronophthisis (Otto et al., 2008). In the same mouse model, mice were observed to have an abnormal interaction between Nek8 and the polycystin complex (Valkova et al., 2005). During the transition from the cell cycle stage to ciliogenesis, NEK8 is activated and then ultimately degraded (Zalli et al., 2012). Mutations in NEK8, including missense and loss-of-function mutations, affected the regulation of signaling in the Hippo pathway through its main effector, YAP (Grampa et al., 2016).

NEK9 has roles in cell division as studies link phospho-NEK9 with microtubule and centrosomal organization, cytokinesis, and centrosome maturation (Roig et al., 2002; Roig et al., 2005; Sdelci et al., 2012; Meirelles et al., 2014). NEK9 has been implicated in the pathologies of Perthes disease, upward gaze palsy, arthrogryposis, congenital contracture syndrome, fetal akinesia, skeletal ciliopathies, meningiomas, and nevus comedonicus (Casey et al., 2016; Levinsohn et al., 2016; Dunn et al., 2019; Abraham et al., 2022; Liu et al., 2022). Ciliogenesis is bidirectionally regulated by selective autophagic cellular processes, whereby ciliogenesis promotes autophagy and vice-versa. NEK9 has been shown to have a role in upregulating autophagy via its binding to ATG8 proteins, which regulate autophagy (Behrends et al., 2010). ATG8 proteins are regulated via a domain on NEK9 called LC3 interacting region (LIR). Negative regulators of autophagy in the ATG8 family include GABARAP and GABARAPL1. Finally, autophagy of NEK9 is required for cilia formation, as NEK9 regulates ciliogenesis by interacting with autophagy adaptor MYH9 and myosin IIA, a suppressor of ciliogenesis (Yamamoto et al., 2021).

NEK10 has a role in promoting optimal cilia length during post-mitotic cilia assembly via interactions with pericentriolar matrix protein 1 (PCM1). NEK10 also stimulates ciliary transport as well as ciliary number and structure (Chivukula et al., 2020). Removal of NEK10 led to a decline in ciliated cells and a decrease in NEK10-promoted cilia resorption (Al Mutairi et al., 2020). Loss-of-function mutations in NEK10 resulted in PCD in humans (Al Mutairi et al., 2020). NEK10 forms a trimeric complex with PCM1 and RIIβ. A protein complex that includes NEK10 is found at centriolar satellites, and the role of NEK10 in this complex makes it essential for ciliogenesis (Porpora et al., 2018).

**FIGURE 4:**
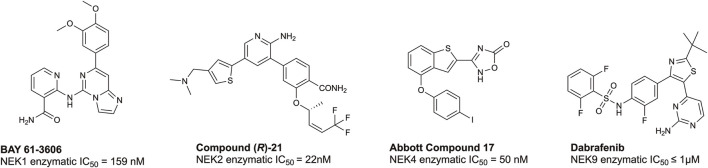
Structures and available potency data for promising chemical leads for NEK1, NEK2, NEK4, and NEK9.

According to the chemical probes portal and SGC databases there are no high-quality chemical probes targeting any member(s) of the NEK family. To date, there are several lead compounds, sourced from the literature, that represent candidates for optimization. Examples of potential tool molecules are summarized in Figure 4. BAY 61-3606 was originally reported as a SYK inhibitor (Yamamoto et al., 2003), but interesting biological activity led to a deeper dive into its kinase target profile, revealing that NEK1 was also a target, with a IC_50_ of 159 nM (Lau et al., 2012). NEK2 is the most well-studied member of the NEK family, and, as such, there are several inhibitors available, including CRUK ICR compound (*R*)-21 with a NEK2 IC_50_ = 22 nM (Innocenti et al., 2012; Wells et al., 2018). This compound shows good selectivity over PLK1, an often seen off-target of NEK2 inhibitors. Some additional NEK2 inhibitors can be found in a NEK-family review (Wells et al., 2018). Abbott compound 17 was originally identified as a MAP3K8 inhibitor (George et al., 2008), and subsequently was found to bind to NEK4 with an IC_50_ = 50 nM (Metz et al., 2011). No further structure–activity relationship (SAR) studies on this scaffold for NEK4 have been reported. The BRAF inhibitor dabrafenib has been approved by the FDA for melanoma. Because dabrafenib showed activity against some cancer lines that differentiated it from other BRAF inhibitors, further kinase profiling was performed. These experiments demonstrated useful and consequential inhibition of CDK16 and NEK9 (Phadke et al., 2018). While some of these candidates bind to their associated NEK with high affinity, the modest kinome-wide selectivity of these compounds has thus far precluded them from being considered chemical probes (Arrowsmith et al., 2015). Through informed SAR campaigns, it should be possible to refine these leads into chemical probes, which may prove to be valuable tools in understanding the role of the NEKs in ciliary biology.

### 3.3 TTBK family

Tau tubulin kinases are a family of serine/threonine/tyrosine kinases that belong to the larger casein kinase superfamily (CMGC) (Sato et al., 2006; Nozal and Martinez, 2019; Bashore et al., 2023). There are two isoforms of TTBK, tau tubulin kinase 1 and tau tubulin kinase 2, and their kinase domains share 88% identity and 96% similarity. Their catalytic residues, K63 and D164 for TTBK1 and K50 and D141 for TTBK2, are similar; however, their non-catalytic domains are distinct from one another (whole sequence similarity: 63% and 35% identity) (Nozal and Martinez, 2019; Bashore et al., 2023). TTBK2 regulates the initiation of ciliogenesis by acting at the distal end of the mother centriole to remove capping protein CP110 from the mother centriole (Čajánek and Nigg, 2014) and facilitate the recruitment of IFT proteins needed for the subsequent assembly of the ciliary axonemal microtubules (Čajánek and Nigg, 2014; Bashore et al., 2023; Binó and Čajánek, 2023). TTBK2 is not only highly expressed in the granular cell layer, cerebellum Purkinje cells, hippocampus, midbrain, and substantia nigra regions of the brain, but also ubiquitously expressed in most human tissues (Houlden et al., 2007; Bashore et al., 2023). As TTBK2 is essential for initiating cilia assembly, it is not surprising that a pathogenic mutation of *TTBK2* leads to a neurological disorder known as spinocerebellar ataxia 11 (SCA11), characterized by atrophy of the Purkinje cells of the cerebellum (Bowie et al., 2018; Bashore et al., 2023). This truncated gene product is lacking the C-terminus, which is involved in trafficking TTBK2 to the basal body of primary cilium and also disrupts its interaction with CEP164, a key substrate that leads to initiation of ciliogenesis (Bowie et al., 2018; Bashore et al., 2023). Defects in ciliary assembly, stability, and function are observed in mice carrying the mutated alleles (Bowie et al., 2018). Another study showed cerebellar degeneration, altered intracellular levels of calcium, and loss of VGLUT2^+^ synapses in *Ttbk2* mutated mice (Bowie and Goetz, 2020).

TTBK1 expression is confined to hippocampal, cortical, and entorhinal cortex neurons (Nozal and Martinez, 2019; Bashore et al., 2023). In human pluripotent TTBK2 knockout stem cells, TTBK1 was able to compensate for the loss of TTBK2 and regulate the assembly of primary cilia during neural rosette formation (Binó and Čajánek, 2023). Binó et al. further showed that in a TTBK2 rescue experiment, TTBK1 activity and expression levels increase enough to compensate for the loss of TTBK2 during neural rosette formation (Binó and Čajánek, 2023). Although TTBK1 lacks the C-terminal CEP164-binding domain that directs TTBK2 to the mother centriole, TTBK1 can still phosphorylate the key substrates of ciliogenesis outside of the mother centriole (Binó and Čajánek, 2023). This study brings to light the first indication that TTBK1 is a regulatory kinase of ciliogenesis. Furthermore, TTBK1 is known to act on neuropathic proteins like tau at pathogenically relevant sites and is overexpressed in Alzheimer’s disease (AD) with single nucleotide polymorphisms associated with late-onset AD (Ikezu and Ikezu, 2014; Bashore et al., 2023). TTBK1 also co-localizes with other neuropathic proteins and neurofibrillary tangles associated with amyotrophic lateral sclerosis and frontotemporal dementia (Ikezu and Ikezu, 2014; Bashore et al., 2023).

**FIGURE 5:**
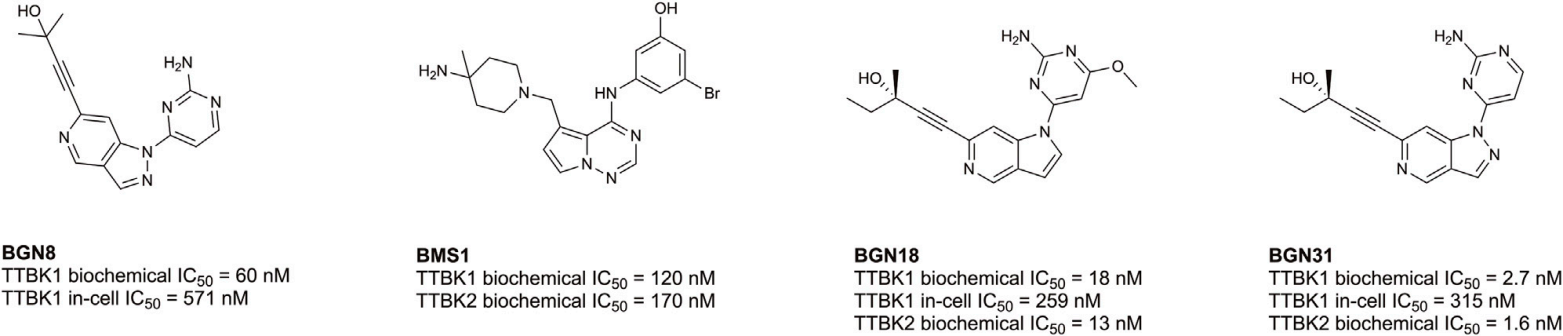
Structures and available potency data for published TTBK1/2 inhibitors.

Despite being understudied, recent efforts have been made towards developing inhibitors of TTBK1/2. Biogen, Bristol-Myers Squibb, and AstraZeneca have published co-crystal structures of TTBK1 bound inhibitors (AZ1, AZ2, BMS1, and BGN18, Figure 5) (Xue et al., 2013; Kiefer et al., 2014; Halkina et al., 2021; Bashore et al., 2023). In 2013, AstraZeneca reported TTBK1 inhibitors AZ1 and AZ2, with Kd values of 0.24 µM and 4.1 µM, respectively (Xue et al., 2013). Later, a group optimized this same scaffold to yield compound 29, with selectivity for TTBK1 over TTBK2 (TTBK1 IC_50_ = 0.24 µM, TTBK2 IC_50_ = 4.22 µM) (Figure 5) (Nozal et al., 2022). Compound 29 was brain penetrant and lowered levels of phosphorylated TDP-43 *in vitro* and *in vivo* in a TDP-43 transgenic mouse model (Nozal et al., 2022). In 2014, Bristol-Myers Squibb published BMS1 and co-crystallized it in complex with TTBK1 (Kiefer et al., 2014). This compound has cell-free IC_50_ values for TTBK1 of 120 nM and for TTBK2 of 170 nM (Kiefer et al., 2014). In 2021, Biogen published a series of brain penetrant, azaindazole-based TTBK1/2 inhibitors: BGN8, BGN18, and BGN31 (Figure 5) (Halkina et al., 2021). Azaindazole BGN8 had a TTBK1 biochemical IC_50_ = 60 nM and in-cell TTBK1 IC_50_ = 571 nM (Halkina et al., 2021). This compound was further optimized to yield BGN18, which demonstrated TTBK1/2 biochemical IC_50_ values of 13–18 nM and TTBK1 in-cell potency of 259 nM (Halkina et al., 2021). Finally, the campaign yielded an azaindole-based analog, BGN31, which is the most advanced inhibitor of the series (Halkina et al., 2021). This compound has single-digit nM TTBK1/2 biochemical IC_50_ values and displays TTBK1 in-cell potency of 315 nM (Halkina et al., 2021). BGN31 was advanced to *in vivo* studies of tau phosphorylation, utilizing a mouse model of hypothermia and a developmental rat model, and was shown to reduce phosphorylation of tau at disease relevant sites (Halkina et al., 2021; Bashore et al., 2023).

The results of an academic effort by the SGC at the University of North Carolina to produce TTBK1/2 inhibitors was published in 2023 (Bashore et al., 2023). The indole scaffold of these inhibitors was discovered from an investigation of the off-target activity of a published Amgen NF-κB inhibitor, AMG28, which included inhibition of TTBK1/2 (Bashore et al., 2023). Compounds 9 and 10 were the most advanced leads from that campaign and these analogs demonstrated TTBK1/2 enzymatic IC_50_ values of 384 nM and 175 nM, respectively, for compound 9 and TTBK1/2 enzymatic IC_50_ values 579 nM and 258 nM, respectively, for compound 10 (Figure 5) (Bashore et al., 2023). Both compounds have been shown to inhibit ciliogenesis in human pluripotent stem cell-based models and phenocopy what is observed due to genetic editing of *TTBK2* (Bashore et al., 2023; Binó and Čajánek, 2023). The available TTBK1/2 inhibitors in Figure 5 are mostly devoid of isoform selectivity, and many of the inhibitors lack overall kinome-wide selectivity data. The high sequence homology between the kinase domains of TTBK1 and TTBK2 suggests that selectivity between these kinases may be very difficult to achieve. To date, no potent and selective chemical probe for either TTBK1 or TTBK2 exists, but delivery of such a compound will facilitate deconvolution of the importance and roles of these kinases in ciliogenesis, canonical signaling pathways, and disease.

## 4 Conclusion

The importance of cilia and the kinases that aid in their regulation is clear. Many of these kinases fall into the understudied category, including members of the CDKL, NEK, and TTBK families, suggesting that we still may uncover new kinases and uncharacterized roles of kinases in ciliary biology. Continued research dedicated to these essential enzymes and the pathways into which they fit will further our understanding of normal and aberrant ciliary functions in human biology and disease. Since many ciliopathies, cancers, and nervous system disorders are caused in part by dysfunctional ciliary pathways, additional knowledge will move the field closer to a more complete understanding of ciliary function and potential treatments for related diseases. The small molecule tools discussed herein are being developed to better understand the roles of cilia in biology and disease. These pre-clinical chemical tools will aid in the dissection of ciliary networks to pinpoint those nodes that, when inhibited, could result in therapeutic benefit for patients.
